# A bibliometric of research trends in acupuncture for spinal cord injury: Quantitative and qualitative analyses

**DOI:** 10.3389/fneur.2022.936744

**Published:** 2022-09-15

**Authors:** Yi Huang, Kelin He, Dandan Fang, Fengjia Ni, Bei Qiu, Kang Liang, Ruijie Ma

**Affiliations:** ^1^Key Laboratory of Acupuncture and Neurology of Zhejiang Province, The Third School of Clinical Medicine (School of Rehabilitation Medicine), Zhejiang Chinese Medical University, Hangzhou, China; ^2^Department of Acupuncture and Moxibustion, The Third Affiliated Hospital of Zhejiang Chinese Medical University (Zhongshan Hospital of Zhejiang Province), Hangzhou, China

**Keywords:** bibliometrics, acupuncture, spinal cord injury, quantitative analysis, qualitative analysis

## Abstract

**Introduction:**

Spinal cord injury (SCI) is a severe disease of the central nervous system with a very high disability rate that seriously affects the daily life of patients. Acupuncture is one of the rehabilitation therapies that has shown significant efficacy in treating post-SCI complications such as motor disorders, neuropathic pain, and neurogenic bladder. Current studies have focused on the effectiveness and mechanisms of acupuncture for SCI, but no studies are available to analyze the bibliometrics of publications related to this area.

**Methods:**

Publications related to acupuncture for SCI were retrieved from the Web of Science Core Collection for quantitative and qualitative analyses. The quantitative analysis was unfolded in the following six main areas: annual publications, countries, institutions, authors, sources, and keywords. The qualitative analysis section screened out publications with high annual citation rates and categorized them according to the study content.

**Results:**

There were 213 relevant publications, more than half of which were journal articles. The number of publications showed a fluctuating upward trend. China and the United States were hub countries for related publications and had extensive cooperation with other countries. The most relevant author was Yuanshan Zeng from Sun Yat-sen University, China. The efficacy and mechanism of acupuncture for neuropathic pain after SCI was the first research hotspot in this field, and electroacupuncture was the most widely used technique. In the past 5 years, the mechanism of acupuncture to improve the local microenvironment of SCI and promote nerve regeneration had become a new research trend. At the same time, acupuncture had been gradually applied to various complications after SCI and in veterinary medicine.

**Conclusion:**

The findings suggest that research on acupuncture for SCI is still flourishing, and more research on electroacupuncture for promoting nerve repair and regeneration after SCI will be available in the future.

## Introduction

Spinal cord injury (SCI) is often caused by trauma, tumors, and infections, which has a high disability rate and cause enormous psychological stress and heavy economic burdens to the patient's family (1). Primary SCI is mostly improved by surgical decompression and spinal fixation, but secondary SCI due to chemical and mechanical injuries is more problematic (2). Currently, the efficacy of drug treatment for the disease is limited, and the tolerability and safety of emerging therapies such as stem cell transplantation, gene therapy, and biomaterials remain to be unequivocally proven (3, 4). Patients with SCI rely heavily on rehabilitation to promote recovery of limb function and reduce the incidence of complications in the later stages.

According to the traditional Chinese medicine theory, meridians are the channels in which the blood and qi flow, as well as the regulatory system of the body's functions (5). Acupoints are specific locations of the meridian line where qi and blood of Zang-fu organs enter and exit (6). As a rehabilitation therapy for SCI, acupuncture can free meridians and harmonize yin and yang by stimulating specific acupoints on the body's surface (7). Clinical practice has confirmed that acupuncture has significant efficacy in post-SCI complications such as motor and sensory impairment, neuropathic pain (NP), and neurogenic bladder (8–10). Fundamental studies have also shown that acupuncture can inhibit apoptosis after SCI, improve the local microenvironment, and promote nerve repair and regeneration (11, 12).

Bibliometrics is a cross-cutting science that applies mathematics and statistics to the quantitative analysis of published scientific literature to measure the impact, interrelationships, and trends of publications in a certain field (13). Currently, bibliometrics has been gradually applied in the fields of medicine, law, education, biology, sports, and other fields (14). Meanwhile, some reviews of acupuncture for SCI have emerged, such as the mechanism of acupuncture with stem cell transplantation to improve neurological function after SCI, acupuncture for SCI complications, systematic review, and meta-analysis of acupuncture and SCI neurological function, acupuncture for NP after SCI (15–18). However, many reviews of acupuncture for SCI have examined only one complication (10, 15, 19), and there are few recent studies. Up to now, there have been no bibliometric studies related to acupuncture for SCI. This study combines quantitative and qualitative analyses of the literature to explore future research directions and goals of acupuncture for SCI.

## Materials and methods

All data were obtained from the Web of Science (WOS) core database, retrieved on March 13, 2022. According to the established study protocol, the search formula was Ts = [(“spinal cord injury” OR “spinal cord injuries”) AND (“acupuncture” OR “electroacupuncture” OR “warming needle moxibustion” OR “fire needling” OR “fire needle” OR “fire acupuncture” OR “acupuncture therapy”)], spanning the period from 1979 to 2021. Two reviewers independently reviewed the titles and abstracts of the above records. Any disagreements that arose during the screening process were resolved through discussion between the two reviewers or by consulting a third reviewer if necessary. Finally, 213 publication records were screened that fit the theme of acupuncture for SCI. Extracted data included reference type, title, journal, publication date, author name and affiliation, and abstract. More details about the search strategy can be found in the first part of the Supplementary material.

Quantitative analysis of the search results was conducted using R software (version 4.1.2) and the Bibliometrix package (20). Complete details are available in Part II of the Supplementary material. Then, among the 213 records, 25 publications with an annual citation rate of no <2.5 were screened for qualitative analysis. Those papers with a high annual citation rate were divided into three groups based on their content: reviews, experimental research papers, and clinical research papers. In the next section, we would provide an in-depth analysis of the collected publication records.

## Quantitative analyses

On March 13, 2022, there were 213 publications related to acupuncture for SCI in the WOS core repository. The information was then analyzed quantitatively in six areas: publications, countries, institutions, authors, journals, and keywords.

### Annual publication analysis

**Figure 1:**
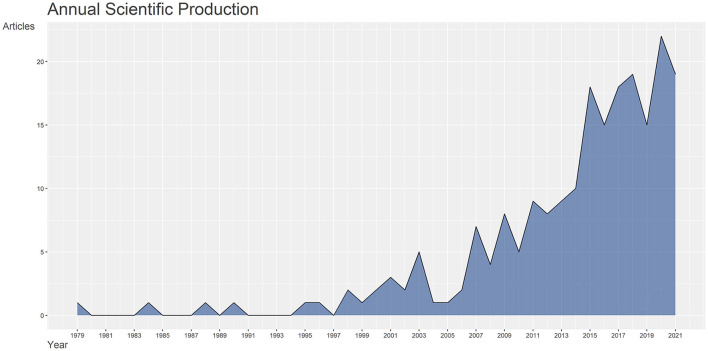
1979–2021 Annual scientific production in the field of acupuncture for SCI.

There were 213 publications from 1979 to 2021 in nine document types, namely articles, reviews, editorial material, meeting abstracts, proceedings papers, letters, corrections, notes, and early access. Of these, journal articles were 159, accounting for 74.65% of the total literature, followed by reviews with 38 articles, as shown in Table 1. Since the late 1970s, the number of annual publications in this field has shown a fluctuating upward trend, as illustrated in Figure 1.

**Table 1:** Central information from publications related to acupuncture for SCI.

**Primary information**	**Results**
Period	1979–2021
Documents	213
Average years from publication	8.22
Average citations per document	17.87
Average citations per year per document	1.77
Document types	9
Authors	890
Single-Authored documents	7
Multi-Authored documents	883
Authors per document	4.18
Co-Authors per document	5.69

**Figure 2:**
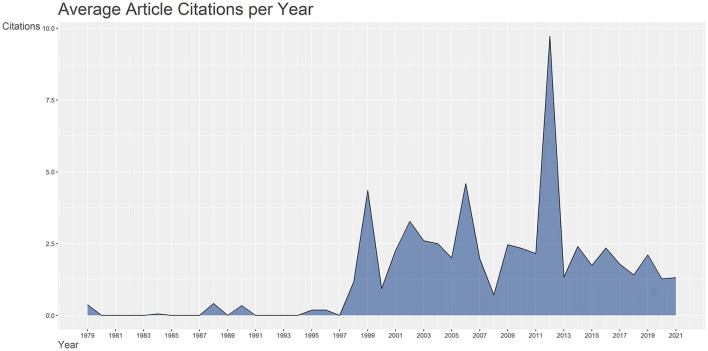
1979–2021 Average article citations per year in the field of acupuncture for SCI.

The total number of citations of these 213 documents was 1951, and the specific average annual article citation rate is shown in Figure 2. Since the advent of publications on acupuncture for SCI in the late 1970s, the average annual article citation rate went through three periods. Before 1997, the number of articles was limited, and the average annual article citation rate trended low and flat. The annual average article citation rate fluctuated upward from 1998 to 2012 and peaked at 9.7 in 2012. The most cited literature, also published this year, was a review of laser acupuncture for critical illnesses such as SCI, brain injury, and heart disease, with a total of 610 citations (21). From 2013 to date, the average annual article citation rate trend had leveled off and stabilized at 1.74/year.

### Analysis of countries

**Figure 3:**
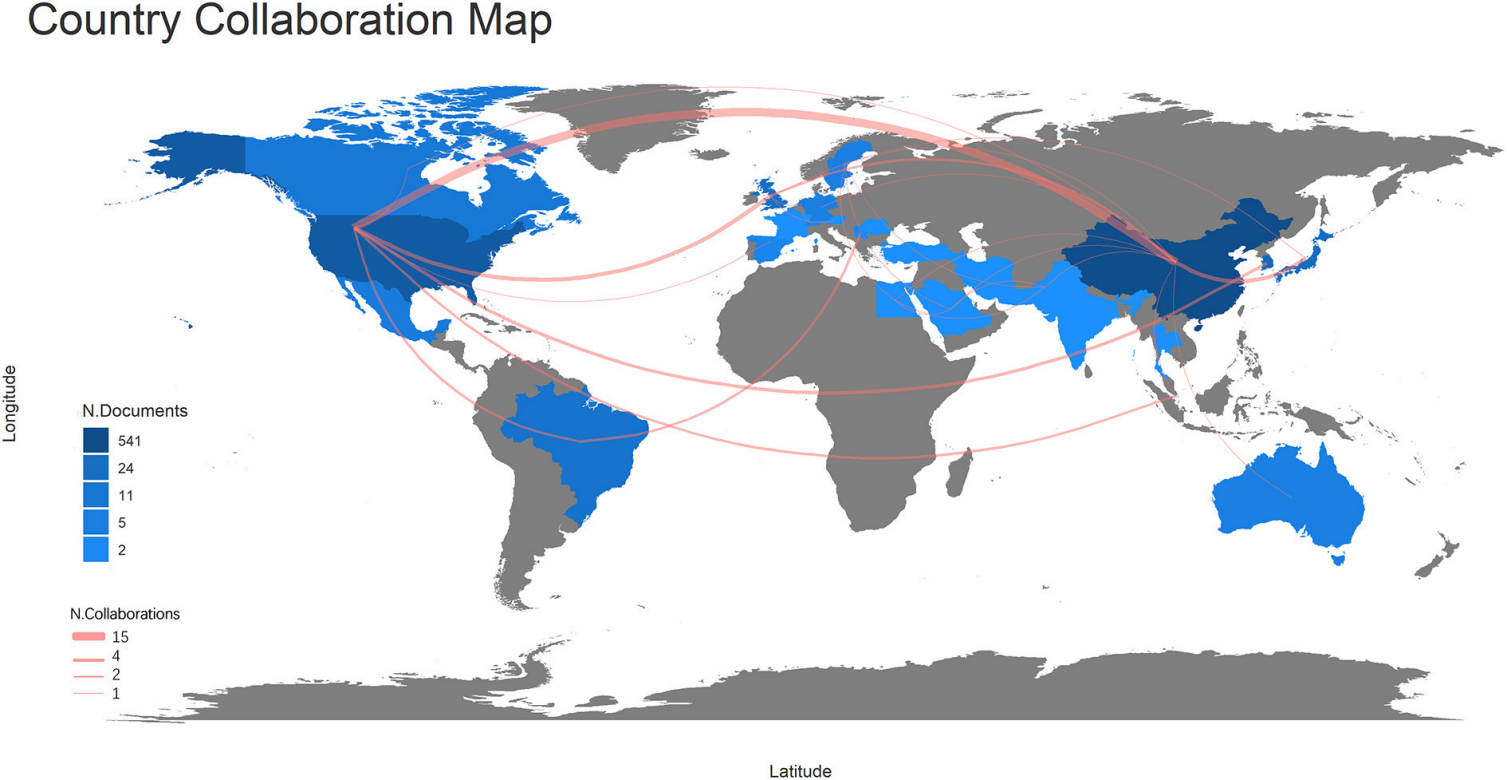
Map of national collaborations for acupuncture for SCI publications (Gray, no relevant publications in the country; blue, related publications in the country; depth of blue, number of publications; red line, connected countries have cooperation in the field, the thickness of the line: frequency of collaboration).

Among the 213 publications, the corresponding authors were distributed in 20 countries or regions, as shown in Figure 3. China has the highest number of publications, accounting for 57.90% of the total, followed by the United States (U.S.) and South Korea. Not surprisingly, the cooperation is pivoted in the U.S. and China and dispersed to other countries or regions, such as Japan, the United Kingdom, and Brazil. In addition, the closest collaboration is between U.S. and China, with 15 collaborative publications.

Table 2 shows that Switzerland, the U.S., and the United Kingdom all have an average article citation rate above 40, which is significantly better than other countries or regions. China, Korea, and Brazil are among the top publishers with low annual article citation rates, with China's average article citations being only 9.56.

**Table 2:** Average article citations for major participating countries.

**Country**	**Average article citations**
USA	45.65
China	9.56
Korea	27.08
Japan	30.62
United Kingdom	40.00
Canada	31.20
Brazil	15.12
Switzerland	46.00
Sweden	29.33
Australia	25.00

### Analysis of institutions

In terms of research institutions, Sun Yat-sen University (19.58%) had the most publications, followed by Beijing University of Traditional Chinese Medicine (8.39%) and Kyung Hee University (8.04%). Seven of the top 10 institutions are from China, while three are from South Korea. See Table 3 for more details.

**Table 3:** Abbreviations and publications of the major participating institutions.

**Affiliations**	**Abbreviations**	**Counts**
SunYat-sen University	SUNYATSENUNIV	56
Beijing University of Chinese Medicine	BEIJINGUNIVCHINESEMED	24
Kyung Hee University	KYUNGHEEUNIV	23
Shanghai Jiao Tong University	SHANGHAIJIAOTONGUNIV	17
Kunming Medical University	KUNMINGMEDUNIV	14
Capital Medical University	CAPITALMEDUNIV	13
Jilin university	JILINUNIV	13
Pusan National University	PUSANNATLUNIV	13
Seoul National University	SEOULNATLUNIV	11
Zhejiang University of Traditional Chinese Medicine	ZHEJIANGCHINESEMEDUNIV	11

### Author Analysis

**Figure 4:**
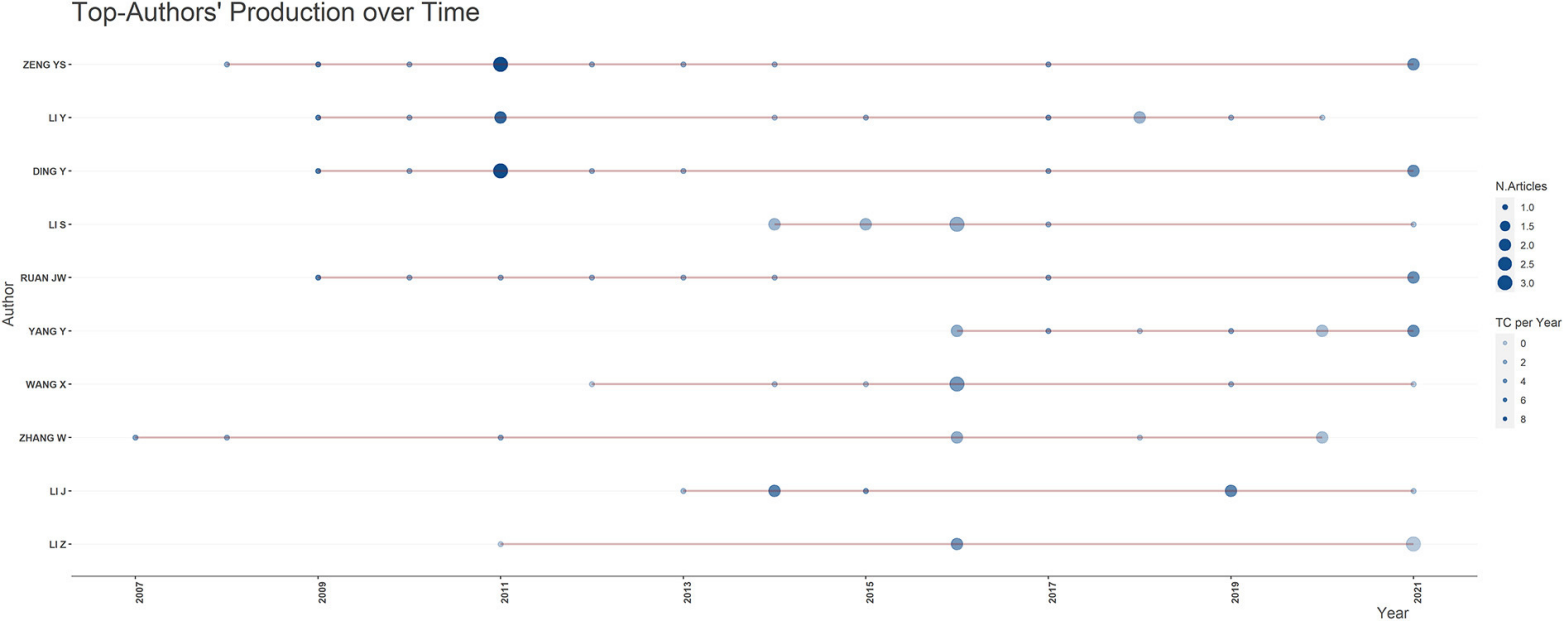
Publications of the top 10 authors in the field of acupuncture for SCI. (red line, timeline of relevant publications by this author; diameter of circle, number of publications; the shade of blue, total citation per year).

In the list of authors of the 213 selected publications, 890 relevant authors were listed. One of the most relevant authors is Yuanshan Zeng from Sun Yat-sen University, China, with 12 publications on acupuncture for SCI, as shown in Figure 4. Not surprisingly, in the last decade, an increasing number of fellows have become involved in the study of acupuncture for SCI.

**Figure 5:**
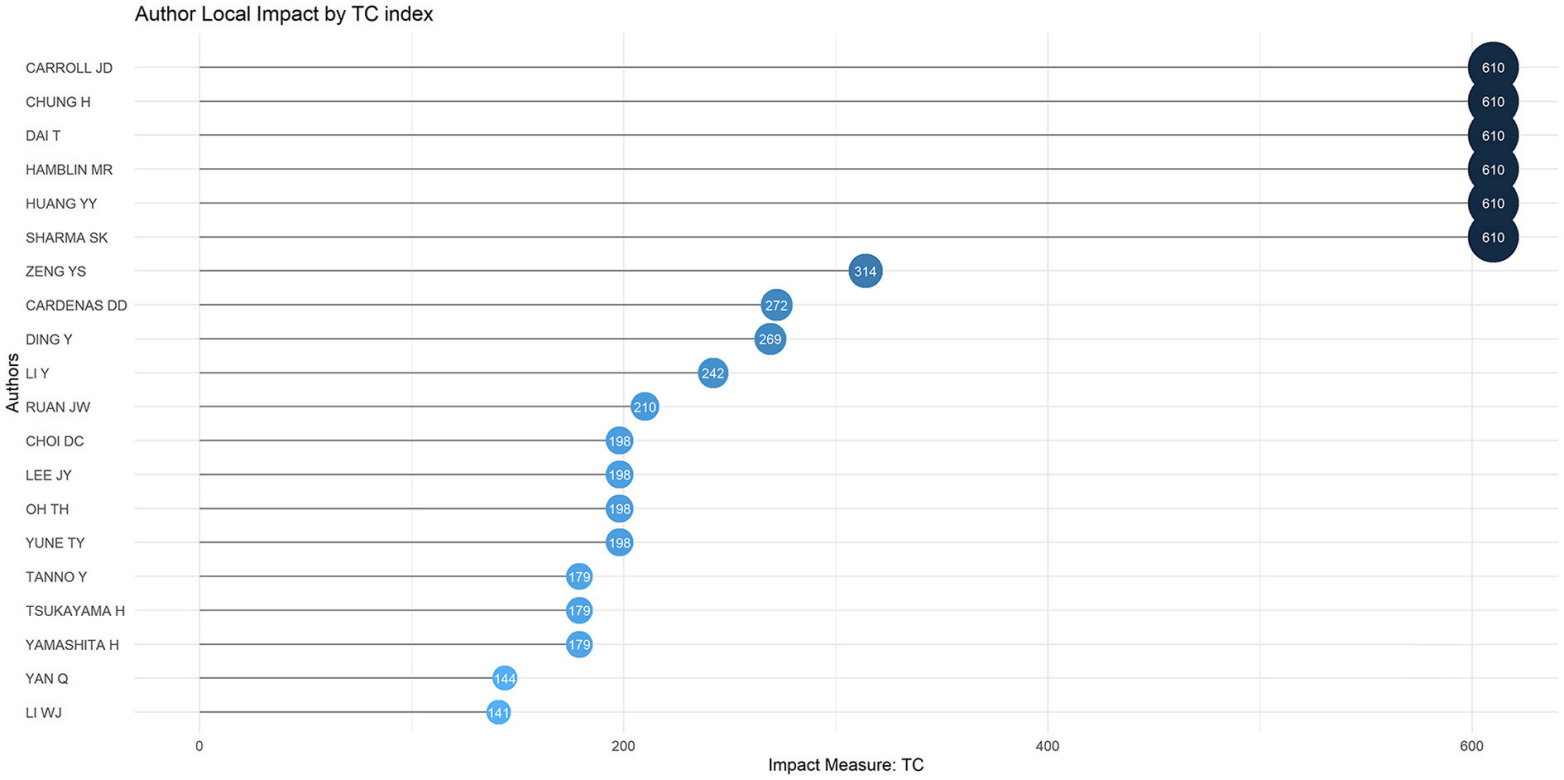
Author local impact on acupuncture for SCI.

The Author's Local Impact can be assessed using the Total Citation Index, as shown in Figure 5. Six authors were cited 610 times, all from Massachusetts General Hospital, USA. However, Yuanshan Zeng, who had the highest number of publications, ranked relatively low, in seventh place, with an overall citation index of 314.

### Sources analysis

**Figure 6:**
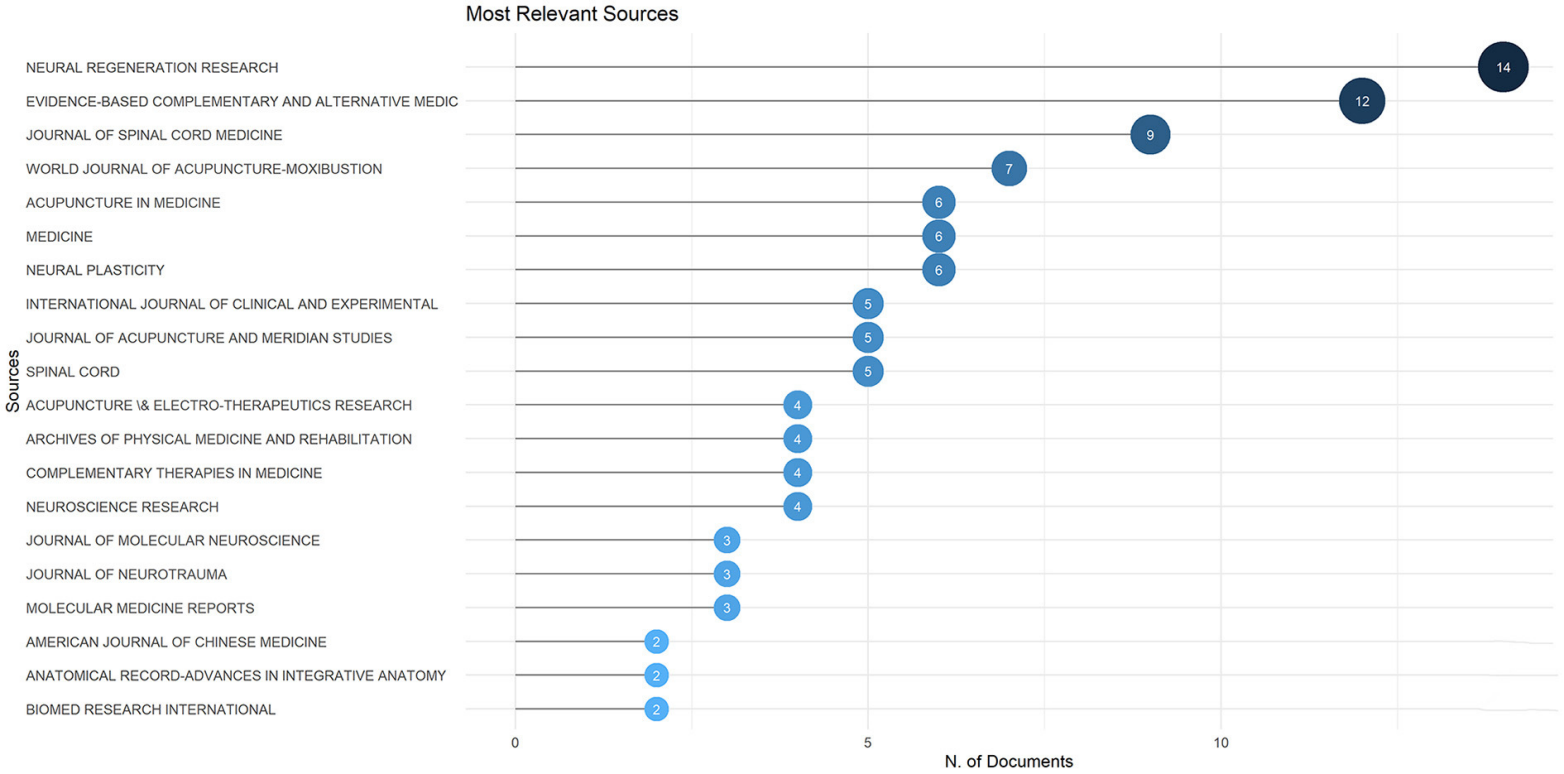
The most relevant sources in the field of acupuncture for SCI.

The top 20 publication sources of acupuncture for SCI were all journals, consistent with more than half of the publications being articles, as shown in Figure 6. “Neural Regeneration Research” published the most articles in this field, with 14 scholarly articles. “Evidence-Based Complementary and Alternative Medicine” ranked second with 12 publications in this field. Most periodicals are in the direction of Chinese medicine or neuroscience, with oncology, rehabilitation, and complementary medicine also covered. The research focus of these journals is critical to selecting target journals for researchers in the field of acupuncture for SCI.

### Keywords analysis

**Figure 7:**
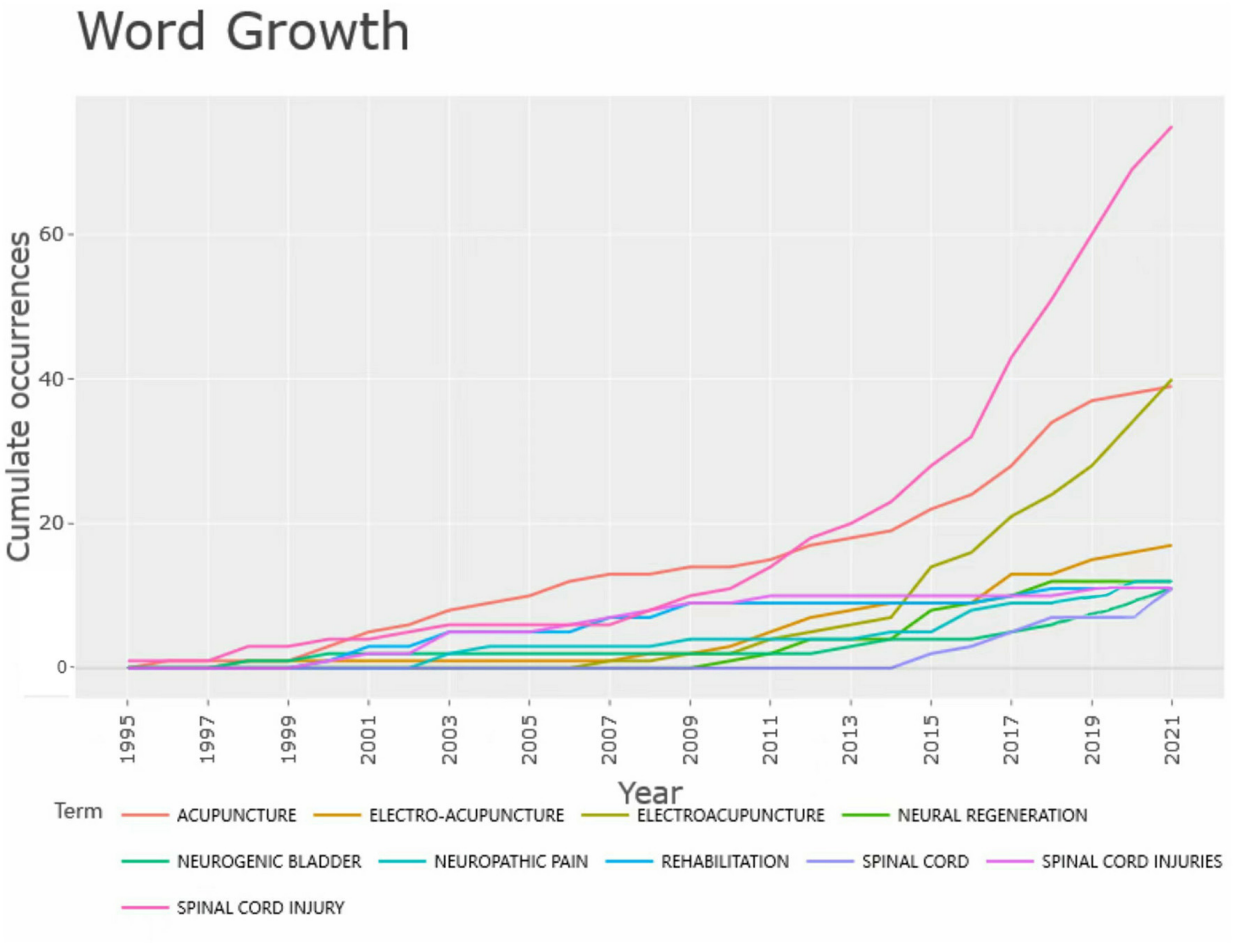
Trends (with Loess Smoothing) in the top 10 author keywords in acupuncture for SCI.

Table 4 shows that “spinal cord injury,” “acupuncture,” and “electroacupuncture” are the three most common keywords in publications on acupuncture for SCI, representing the main scope of the study. The key words “neuropathic pain,” “neurogenic bladder” and “rehabilitation” represent the superior diseases of acupuncture SCI. The use of the keywords “spinal cord injury,” “acupuncture,” and “electroacupuncture” has increased rapidly over the past decade, as shown in Figure 7. This trend not only indicates a growing number of articles in the field but also points to the superior technique of acupuncture for SCI, namely electroacupuncture (EA).

**Table 4:** Top 10 author keywords in the field of acupuncture for SCI.

**Words**	**Occurrences**
Spinal cord injury	76
Acupuncture	40
Electroacupuncture	40
Electro-Acupuncture	17
Neural regeneration	12
Neuropathic pain	12
Neurogenic bladder	11
Rehabilitation	11
Spinal cord	11
Spinal cord injuries	11

**Figure 8:**
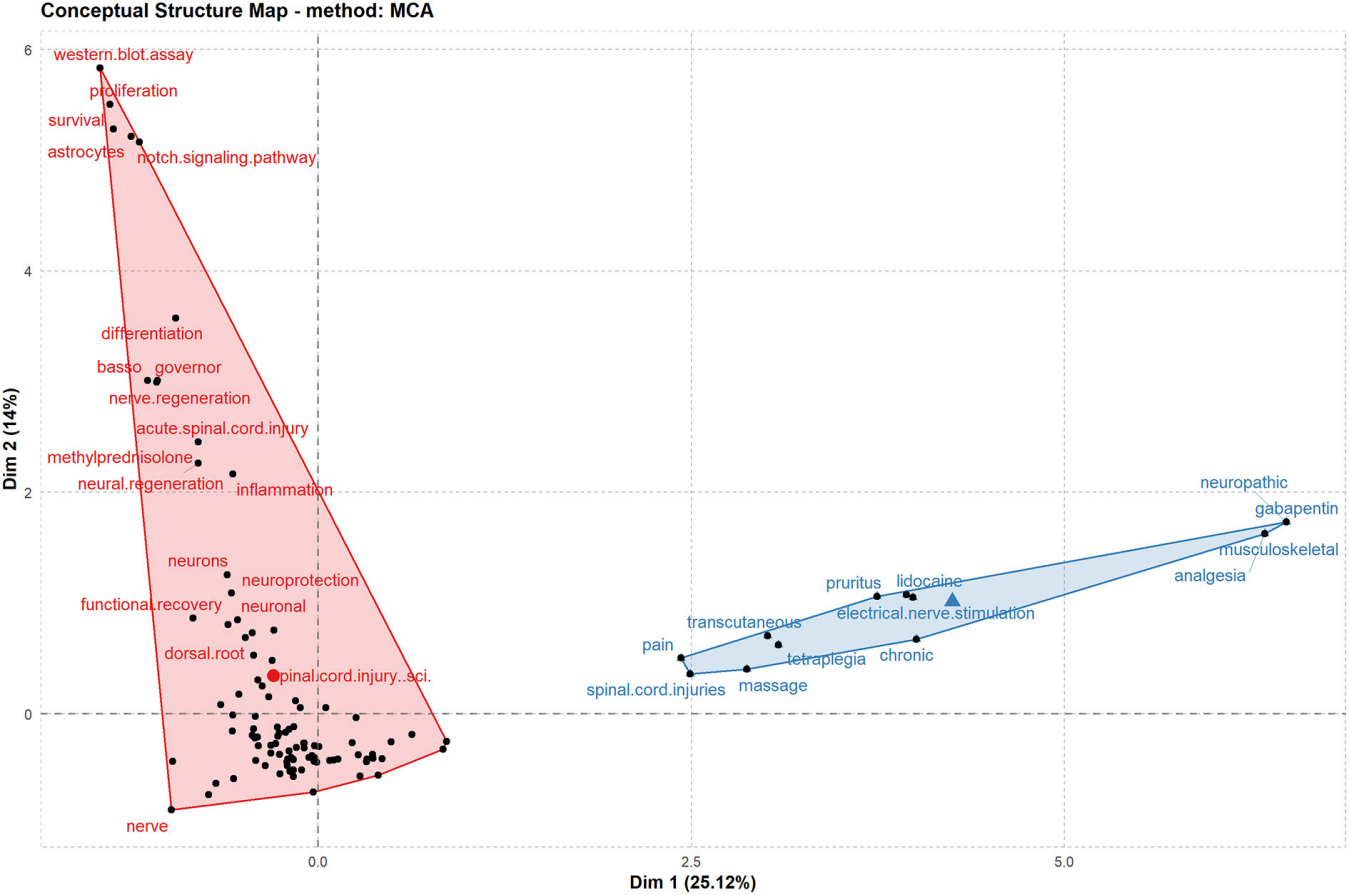
Conceptual structure map of common author keywords in acupuncture for SCI (Multilateral graphics: topic clusters, keywords in the same cluster have similarity).

Figure 8 represents the author's keywords as a two-dimensional planar diagram using multiple correspondence analysis, called the concept structure diagram. Multiple correspondence analysis is an exploratory multivariate technique for the graphical and numerical analysis of multivariate categorical data. The figure divides the author keywords into two clusters, with red clusters focusing on functional recovery after SCI and blue ones on NP after SCI. The chart gives the reader a quick and general overview of the research hot spots in the field.

## Qualitative analyses

From 1979 to 2021, there were 213 publications in the field of acupuncture for SCI in the WOS core database. The annual citation rate is calculated by dividing the total number of citations by the number of years since publication, and publications with high annual citation rates are usually more valuable (22). This data set was sorted according to the annual citation rate from highest to lowest, and publications with an annual citation rate more than or equal to 2.5 were included, resulting in 25 publications, accounting for 11.74% of the total. Qualitative analysis of the literature helps scholars find research directions in acupuncture for SCI that deserves further attention.

**Figure 9:**
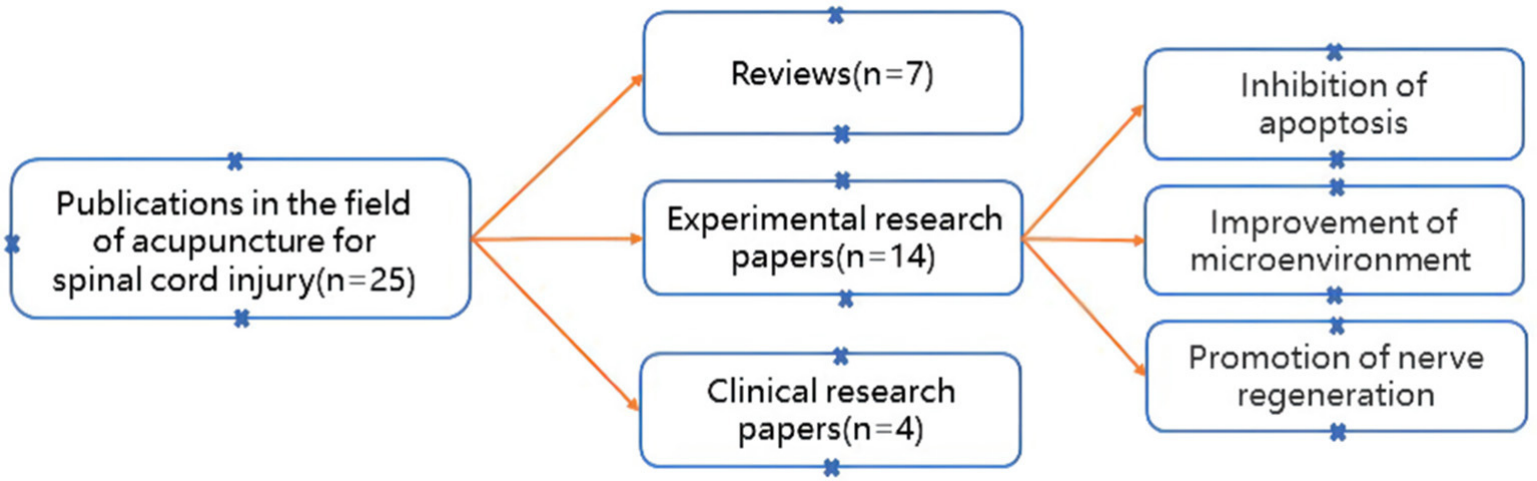
Classification of publications (annual citation rate ≥2.5) for acupuncture treatment SCI.

Figure 9 shows the specific classification of these 25 publications with an annual citation rate of more than 2.5. The first category is the review, which contains one Meta-analysis. The second category is experimental research papers, which are further divided into three subcategories according to specific research directions: inhibition of apoptosis, improvement of the microenvironment, and promotion of nerve regeneration. The third category is clinical research papers, including subjects of patients and animals with SCI. The details of these categories are described in Section Reviews analysis, Experimental research papers analysis, and Clinical research papers analysis. In addition, the annual citation rates, DOI numbers, and types of these papers are shown in Table 5.

**Table 5:** Publications with annual citation rates more than or equal to 2.5 and their categories.

**Rank**	**Annual citation rate**	**Paper**	**Doi**	**Category**
1	55.455	Chung H. 2012, Ann Biomed Eng	10.1007/s10439-011-0454-7	1
2	14	Sawynok J, 2016, Neuroscience	10.1016/j.neuroscience.2015.10.031	1
3	9.625	Da Silva MD, 2015, Mol Neurobiol	10.1007/s12035-014-8790-x	2 (2)
4	8.778	Boldt I, 2014, Cochrane Database Syst Rev	10.1002/14651858.CD009177.pub2	1
5	8	Choi DC, 2012, Exp Neurol	10.1016/j.expneurol.2012.05.014	2 (2)
6	5	Hong E, 2021, J Spinal Cord Med	10.1080/10790268.2019.1665612	2 (3)
7	4.333	El-Seedi HR, 2020, Trends Food Sci Technol	10.1016/j.tifs.2020.04.026	1
8	4	Do Espirito Santo CC, 2019, Brain Behav Immun	10.1016/j.bbi.2019.01.012	2 (2)
9	3.667	Zhao J, 2017, Acupunct Med	10.1136/acupmed-2016-011107	2 (2)
10	3.6	Cai W, 2018, Am J Chin Med	10.1142/S0192415X1850026X	1
11	3.556	Jiang S, 2014, Evid -Based Complement Altern Med	10.1155/2014/431580	2 (2)
12	3.5	Geng X, 2015, Neural Regen Res	10.4103/1673-5374.153687	2 (3)
13	3.143	Glaser J, 2016, Spine	10.1097/BRS.0000000000001525	3
14	3	Estores I, 2017, J Spinal Cord Med	10.1080/10790268.2016.1141489	3
15	3	Zhang YT, 2017, Neural Plast	10.1155/2017/7351238	2 (2)
16	3	Pak ME, 2018, Exp Neurol	10.1016/j.expneurol.2017.11.014	2 (3)
17	2.9	Lee JY, 2013, PLoS ONE	10.1371/journal.pone.0073948	2 (3)
18	2.833	Liu J, 2017, Biomed Pharmacother	10.1016/j.biopha.2017.02.077	2 (1)
19	2.667	Li G, 2020, Neurosci Bull	10.1007/s12264-019-00442-0	2 (3)
20	2.636	Jia J, 2012, J Ethnopharmacol	10.1016/j.jep.2012.01.034	1
21	2.6	Zidan N, 2018, J Neurotrauma	10.1089/neu.2017.5485	3
22	2.5	Zhu XL, 2017, Brain Res	10.1016/j.brainres.2017.01.008	2 (1)
23	2.5	Prado C, 2019, Res Vet Sci	10.1016/j.rvsc.2019.01.011	3
24	2.5	Zhu Y, 2019, Ann Phys Rehabil Med	10.1016/j.rehab.2018.09.010	1
25	2.5	Xu H, 2021, J Neurotrauma	10.1089/neu.2020.7155	2 (3)

Category: 1 review; 2 (1) inhibition of apoptosis; 2 (2) improvement of the microenvironment; 2 (3) promotion of neural regeneration; 3 clinical research papers.

### Reviews analysis

Among the 25 high annual citation publications in acupuncture for SCI, there were seven reviews, accounting for 28% of the total. This percentage was significantly higher than the share of reviews in all publications, 17.84% for the latter. Five of these seven reviews were in the top 10, and the most cited literature in terms of annual citation rate was also a review. Therefore, the reviews are classified as the first category in this field. A chronological qualitative analysis based on these quality reviews can help people to get an overview of the research and to grasp the research trends.

Research related to acupuncture for SCI began in the late 1970s, and only in 2012 was a high annual citation rate review in this area published. The peak of review publication occurred in 2020. A 2012 review on low-level laser therapy summarized the mechanisms of action and indications for low-level laser therapy while describing laser acupuncture stimulation in acupoints for severe diseases such as SCI and stroke (21). The review had attracted significant attention and had been cited 610 times since publication, with an annual citation rate of 55.455, four times that of the second-ranked publication in the field.

In the same year, researchers suggested that SCI had been used as a pilot for functional genomic technology and that combining basic acupuncture research with this technology could help explore the mechanisms involved (23). In 2014 Boldt et al. used a meta-analysis to summarize non-pharmacological interventions for chronic pain in patients with SCI and confirmed the effectiveness of acupuncture in pain relief (10). The high annual citation rate review in 2016 continued the hot issue of acupuncture analgesia. Sawynok concluded that all adenosine receptors modulated nociception through spinal glial cells. In addition, acupuncture could treat NP from SCI by increasing the level of endogenous adenosine (24).

With the widespread use of acupuncture techniques and the advancement of basic research in recent years, the topics of reviews on acupuncture for SCI tended to explore the relevant mechanisms and new clinical indications. In 2018, Cai and Shen investigated the anti-apoptotic mechanisms of acupuncture in neurological disorders such as SCI. The literature suggested that the anti-apoptotic effect of acupuncture was mainly characterized by elevated expression of B-cell lymphoma-2 and decreased expression of Bax and caspases, which might be related to the upregulated expression of a series of downstream signaling pathways and neurotrophic factors (25). In 2019, a study summarized the therapeutic effects and influencing factors of EA on spasticity after upper motor neuron lesions (26).

### Experimental research papers analysis

**Figure 10:**
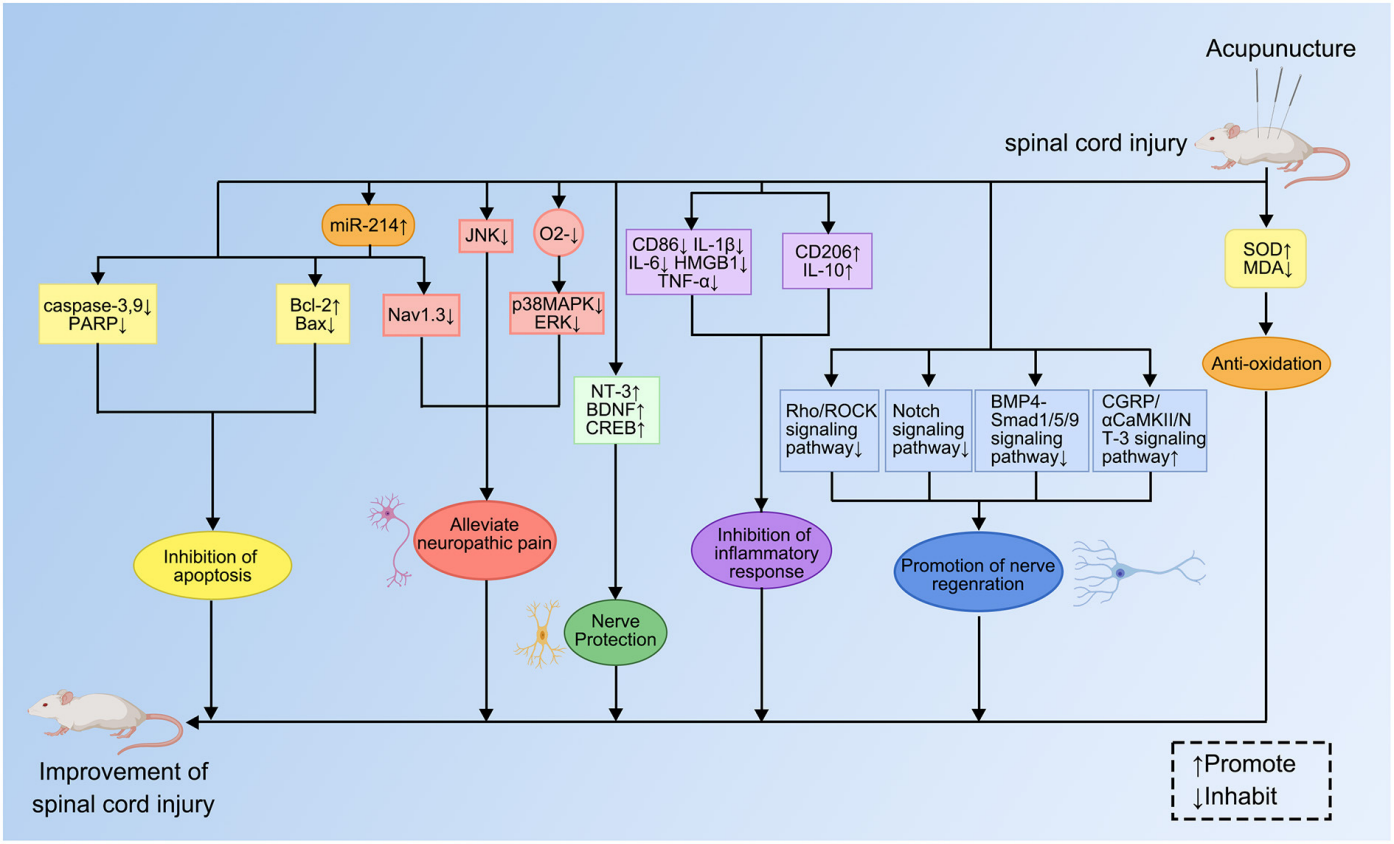
Specific mechanisms of acupuncture for SCI.

This section reviewed publications with high annual citations in the category of experimental studies on acupuncture for SCI. This category had 14 publications, accounting for 56% of the high annual citation rate publications, and spanned from 2012 to 2021. These publications were further divided into inhibition of apoptosis, improvement of the microenvironment, and promotion of neural regeneration according to the research direction, with the specific mechanisms shown in Figure 10. The following is a chronological discussion of the experimental research literature in this field over the past 10 years.

#### Inhibition of apoptosis

In the field of acupuncture for SCI, two publications with high annual citation rates focused on the inhibition of apoptosis, both published in 2017. Apoptosis is an active, programmed process of spontaneous cell disassembly in response to environmental stimulus signals, changes in environmental conditions, or palliative damage (27). It is now believed that apoptosis of a large number of neurons, astrocytes, oligodendrocytes, and microglia in the injured spinal cord is one of the pathological mechanisms of secondary injury after SCI, and inhibition of apoptosis can effectively promote recovery of the SCI (28). Liu and Wu found that EA inhibited the SCI-induced upregulation of the apoptosis-related protein Bax and the pain-related protein Nav1.3 by regulating the expression of mir-214 to achieve analgesia and antiapoptotic effects (11). In the same year, Zhu et al. found that EA preconditioning reduced spinal cord ischemia-reperfusion injury (29).

#### Improvement of the microenvironment

With the in-depth basic research in the past 10 years, the research results on the mechanism of acupuncture to improve the microenvironment of SCI were fruitful. Since 2012, there had been seven high annual citation papers in this field, accounting for half of the high annual citation papers in the experimental research category. Imbalance in the local microenvironment of SCI often leads to hemorrhage or ischemia, secondary to an inflammatory response and the formation of glial scarring that accelerates and exacerbates the injury (30). The mechanisms that improve the microenvironment of SCI specifically include inhibiting inflammatory responses, lipid peroxidation, and excitatory amino acid toxicity, increasing neurotrophic factors and blood flow, and improving microcirculation in several ways (31). A chronological overview of these findings was in the following.

In 2012 Choi et al. found that the relief of NP from SCI by needling Shuigou (GV26) and Yanglingquan (GB34) might be related to the inhibition of p38MAPK and ERK activation and inflammatory mediator release (32). In 2013 Lee et al. similarly selected these two acupoints to study the mechanism of acupuncture analgesia. Acupuncture has been found to alleviate NP, such as mechanical and thermal hypersensitivity, by inhibiting the activation of Jun-N-terminal kinase in astrocytes after SCI (33). In 2014, Jiang et al. found that EA, manual acupuncture, and transcutaneous acupoint electrical stimulation all had antioxidant, anti-inflammatory, and anti-apoptotic effects (34). Macrophages are classified into M1 and M2 subtypes based on surface markers. M1-type macrophages promote immune responses by increasing phagocytosis and releasing pro-inflammatory factors. In contrast, M2-type macrophages suppress inflammatory responses and promote tissue repair (35). In 2015 da Silva et al. found that manual acupuncture of Sanyinjiao (SP6) induced a phenotypic switch in muscle macrophages with a decrease in M1 macrophages and an increase in M2 macrophages and IL-10 in muscle to reduce pain, edema, and inflammation (36).

Neurotrophin-3 (NT-3) contributes to neuroprotection and axonal regeneration (37). There were two high annual citation papers on acupuncture increasing NT-3 expression in 2017. Zhao et al. found that EA at the acupoint inhibited M1 macrophages, TNF-α, IL-1β, and IL-6 levels, but enhanced the expression of IL-10, M2 macrophages, and NT-3 (38). These views were corroborated by the study of Zhao et al. This study showed that either tail nerve electrical stimulation or EA treatment could increase NT-3 and choline acetyltransferase expression, protect motor neurons, and reduce muscle atrophy in rats with thoracic SCI (39).

A study in 2019 found that SCI promoted anxiety and depression-like behaviors in adult female rats (40). This condition is associated with an imbalance between the production and release of pro- and anti-inflammatory cytokines, which acupuncture can help (41).

#### Promotion of nerve regeneration

Mechanisms that promote nerve repair have been a hot topic of research in acupuncture for SCI over the last few years. There are five related high-quality publications, which were published from 2015 to 2021 and all centered on EA. Nerve regeneration and repair can manifest as axonal lengthening, axonal remyelination, or neural stem cell differentiation (1).

Notch signaling is accomplished by transmembrane ligands on the cell activating transmembrane receptors on just cellular cells, which can regulate cellular transduction and participate in angiogenesis (42). In 2015, Geng et al. found that EA inhibited the Notch signaling pathway, promoted the proliferation of endogenous neural stem cells, and inhibited their differentiation to astrocytes (43).

Demyelination often occurs after oligodendrocyte necrosis and apoptosis and is one of the common pathological findings after SCI (44). A 2018 study discovered that EA in Baihui (GV20) and Zusanli (ST36) combined with treadmill exercise promoted oligodendrocyte production in the corpus callosum of hypoxic-ischemic neonatal rats, induced the expression of mature brain-derived neurotrophic factor, and attenuated demyelination (45). This study corroborated that EA improved demyelination after SCI. Epidural spinal cord stimulation is an alternative electrical stimulation method to EA, in which electrodes are implanted in the epidural space of the spinal cord to stimulate the spinal cord tissue directly for therapeutic effect (46). In 2020, Li et al. found that this therapy enhanced oligodendrocyte survival and differentiation and protected myelin by inhibiting the BMP4-Smad1/5/9 signaling pathway after SCI (47).

In 2021, researchers found that EA in Yaoyangguan (GV3) and Dazhui (GV14) inhibited the Rho/ROCK signaling pathway, promoted axonal regeneration, and reduced inflammatory responses (48). In the same year, a study found that the EA in the governor vessel (*Du Mai*) increased NT-3 expression and activated the intrinsic growth capacity of spinal cord neurons after SCI through the CGRP/RAMP1/L-VGCC/NT-3 pathway, which promoted neuronal survival, axonal regeneration (12).

### Clinical research papers analysis

In addition to the review and the experimental research papers, there are several highly annual cited clinical research papers in acupuncture for SCI, in both human and veterinary subjects, which are discussed together in this section. In 2016 Glaser et al. found that transcranial direct current stimulation helped to reduce the dose of analgesic drugs after SCI (49). In 2017 Estores used auricular acupuncture to treat NP after SCI and showed significantly improved numerical rating scale results compared to the baseline period (50).

The high annual citation publications included two veterinary clinical research papers on acupuncture for SCI (51, 52). The subjects of the two papers were canines with acute SCI functional recovery and chronic SCI. Treatment techniques included a pulsed electromagnetic field and a combination of stem cell and EA therapy with acupoints mainly from the governor vessel and the bladder meridian. Both therapies showed functional improvement in dogs with SCI. However, the sample size of both trials was <20 cases, and further validation is still needed.

## Limitations

First, this bibliometric analysis did not include non-English databases, although the WOS database is reliably sourced and contains chiefly quality publications in acupuncture for SCI. Second, qualitative analysis is subjective to the researcher, and different observation perspectives may lead to different conclusions. Third, the publication of academic research lags behind clinical practice. The results of this study reflect only the academic research trends in the field, being the primary purpose of this bibliometric study.

## Discussion

The study conducted quantitative and qualitative analyses on 213 publications in the field of acupuncture for SCI in the WOS core database from 1979 to 2021.

Quantitative analysis shows a fluctuating upward trend in the number of publications and the average annual citation rate in this area. The authors are mainly from Asian and American countries, with China and U.S. as pivotal countries having extensive collaboration with other countries. China leads the world with more than four times the number of publications as the U.S., but it ranks second to the U.S. in terms of citations. It shows more Chinese authors in the field, but more quality publications come from the U.S. The most relevant author is Yuanshan Zeng from Sun Yat-sen University, China, with 12 publications on acupuncture for SCI. However, his author impact ranking is relatively low at seventh, with the top six coming from Massachusetts General Hospital, USA. The 20 most relevant publication sources are all academic journals, consistent with more than half of the publications being journal articles. Keyword analysis shows that EA has been a hot research topic in recent years.

In the qualitative analysis section, we analyzed 25 publications with an annual citation rate of no <2.5. These publications are divided into three categories based on content, including reviews, experimental research papers, and clinical research papers. A comprehensive analysis of the various publications according to publication time concludes that the efficacy and mechanism of acupuncture for NP after SCI is the first research hotspot, and EA is the most widely used technique. In addition, the most commonly used acupoints for EA are those located at the governor vessel. In the last 5 years, due to intensive basic research, the mechanism of acupuncture to improve the local microenvironment of SCI and promote nerve regeneration has become a new research trend. At the same time, as the efficacy of acupuncture has been generally confirmed, acupuncture is gradually used in various complications after SCI and the veterinary field.

## Conclusion

The article provides an essential reference for understanding and predicting the direction of research in acupuncture for SCI. It is clear that research on acupuncture for SCI is still flourishing, and more research results based on EA to promote nerve repair and regeneration after SCI will be available in the future.

## Data availability statement

The original contributions presented in the study are included in the article/Supplementary material, further inquiries can be directed to the corresponding author.

## Author contributions

YH analyzed the data and edited the manuscript. KH designed the entire research. DF, FN, and BQ were responsible for data collection and participated in revising the manuscript. RM made significant contributions to the analysis and interpretation of the data. All authors read the manuscript and agreed to submit it.

## Funding

This work was supported by the Chinese Medicine Research Program of Zhejiang Province (Grant No. 2019ZZ013), National Natural Science Foundation of China (Grant No. 82174487), Health and Health Innovation Talent Project of Zhejiang Province (Grant No. 2021RC099), and General Research Project of Zhejiang Provincial Education Department (Grant No. Y202044576).

## Conflict of interest

The authors declare that the research was conducted in the absence of any commercial or financial relationships that could be construed as a potential conflict of interest. The reviewer WD declared a shared affiliation with the authors to the handling editor at the time of review.

## Publisher's note

All claims expressed in this article are solely those of the authors and do not necessarily represent those of their affiliated organizations, or those of the publisher, the editors and the reviewers. Any product that may be evaluated in this article, or claim that may be made by its manufacturer, is not guaranteed or endorsed by the publisher.
