# Supplementary material for: A bibliometric of research trends in acupuncture for spinal cord injury: Quantitative and qualitative analyses
Source: Front Neurol. 2022 Sep 15;13:936744. doi: 10.3389/fneur.2022.936744 (PMC9521612; doi:10.3389/fneur.2022.936744)
Supplement: Supplementary file 1 [file Presentation_1.PDF]

Supplementary Material

The supplementary material consists of two parts. The first part is the search strategy, and the second is the software code and the operation of the Bibliometrix package.

Part I. Search Strategies

Step 1: Go to the official Web of Science website and select the Web of Science core collection.

Step 2: Enter the search formula as follows.

Ts= ((“spinal cord injury” OR “spinal cord injuries”) AND (“acupuncture” OR “electroacupuncture” OR “warming needle moxibustion” OR “fire needling” OR “fire needle” OR “fire acupuncture” OR “acupuncture therapy”))

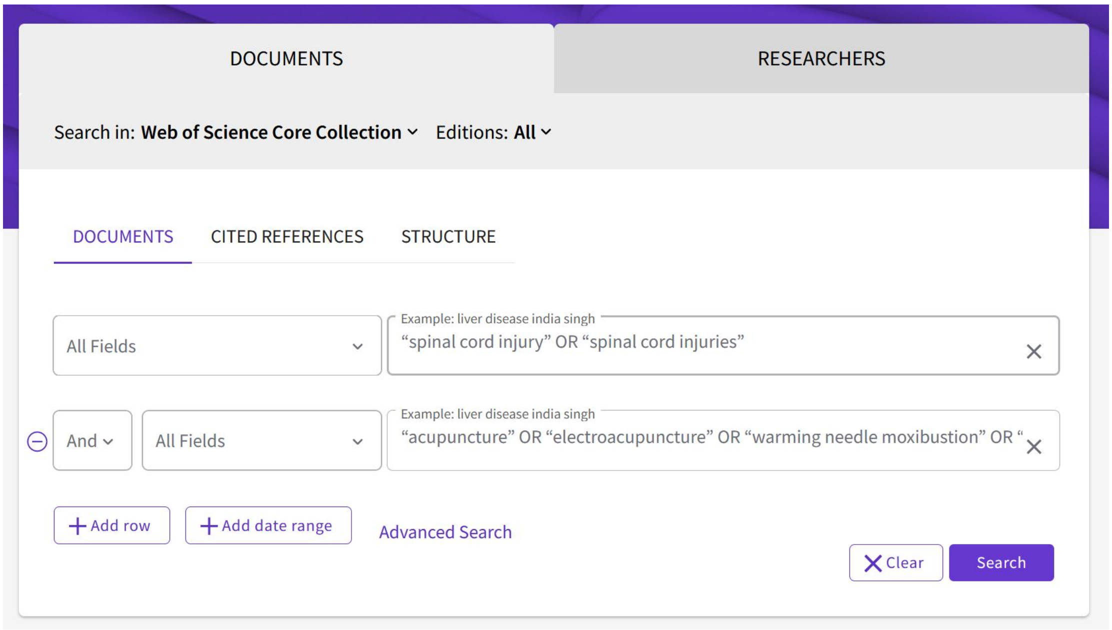

Fig. 1 Schematic diagram of search formula

Step 3: Click on the publication year to exclude 2022.

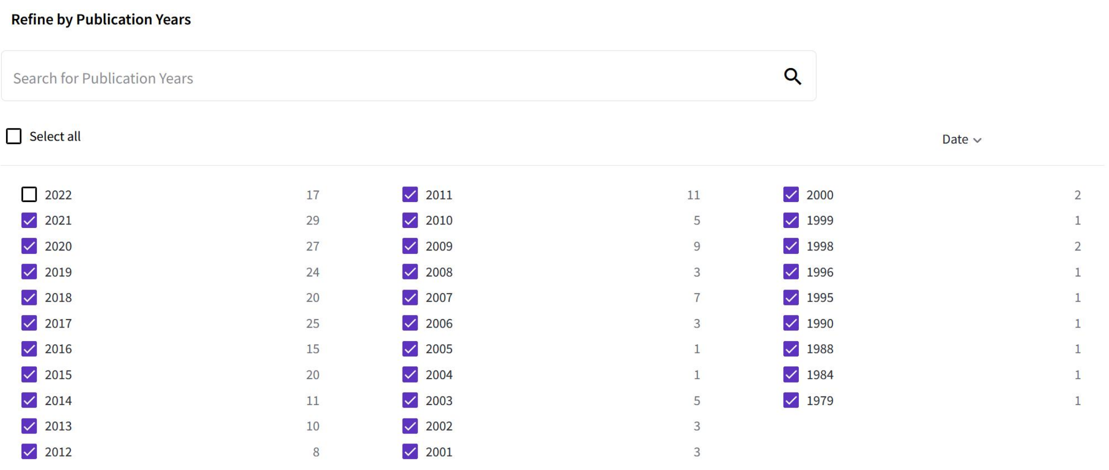

Fig. 2 Selection of the publication year

Step 4: The search results show 251 relevant publication records from 1979-2021.

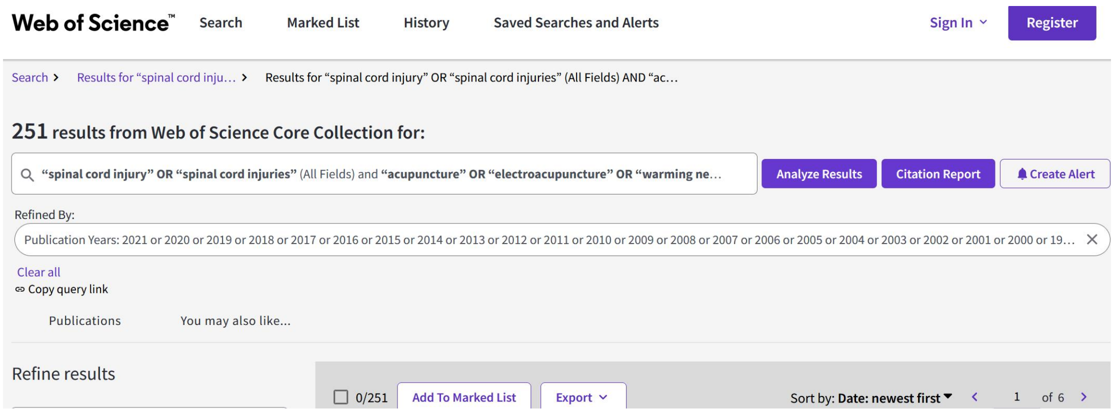

Fig. 3 Search result

Step 5: Two reviewers independently reviewed the titles and abstracts of the above records. Any disagreements that arose during the screening process were resolved through discussion between the two reviewers or by consulting a third reviewer if necessary. Finally, 213 publication records were screened that fit the theme of acupuncture for SCI. Then, 25 publications with high annual citations were screened out of 213 records for qualitative analysis. The following figure shows the data collection and filtering process for the bibliometric analysis of acupuncture for spinal cord injury.

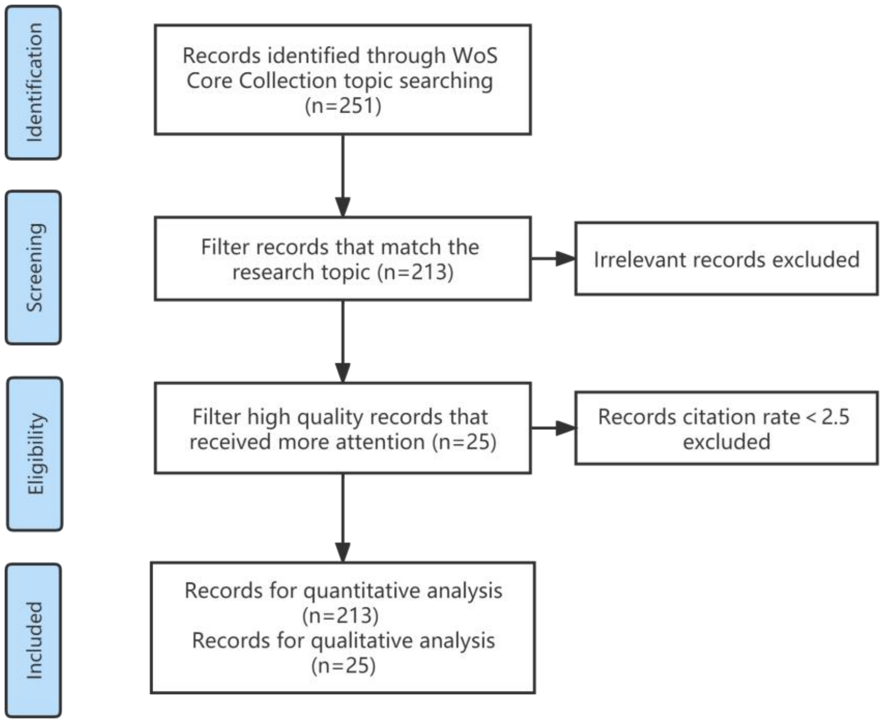

Fig. 4 Flow chart for screening publication records

Part II. Software Code and Operation of Bibliometrix Package

Step 1: Install and import the bibliometrix toolkit in the R software.

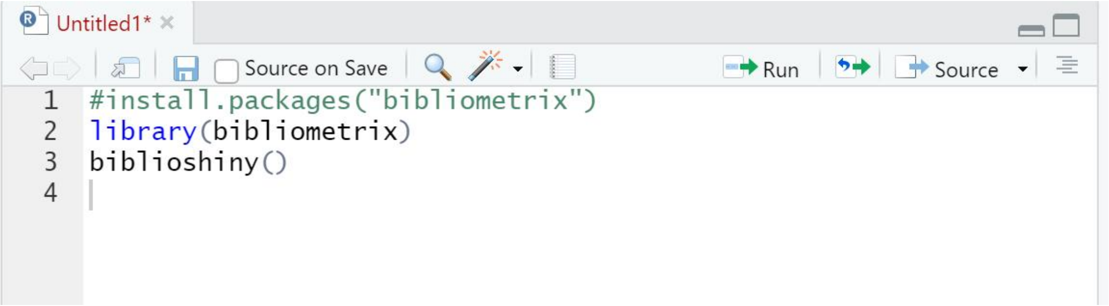

Fig. 1 Software code

Step 2: Open the biblioshiny app (pop up through the default browser) and click on "Load Data".

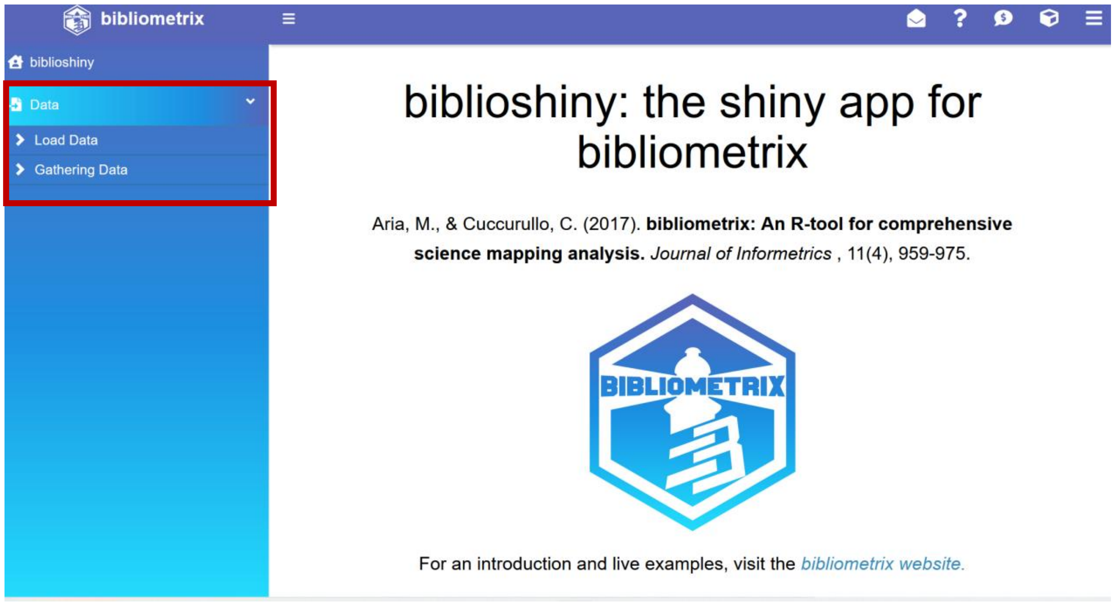

Fig. 2 Home page of the biblioshiny website

Step 3: Import the filtered raw files in the right menu bar, and click the "Start" button to enter the data analysis interface.

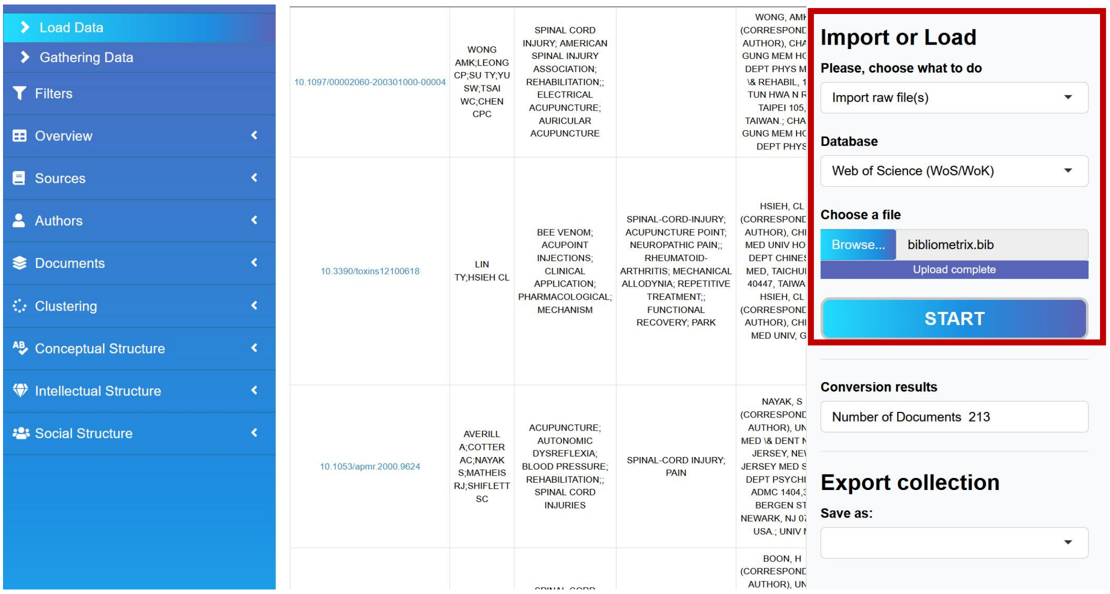

Fig. 2 Import of data

Step 4: The left menu bar contains various options for relevant publication information, such as authors, resources, citation status, etc. The following chart shows the main information of the related publications.

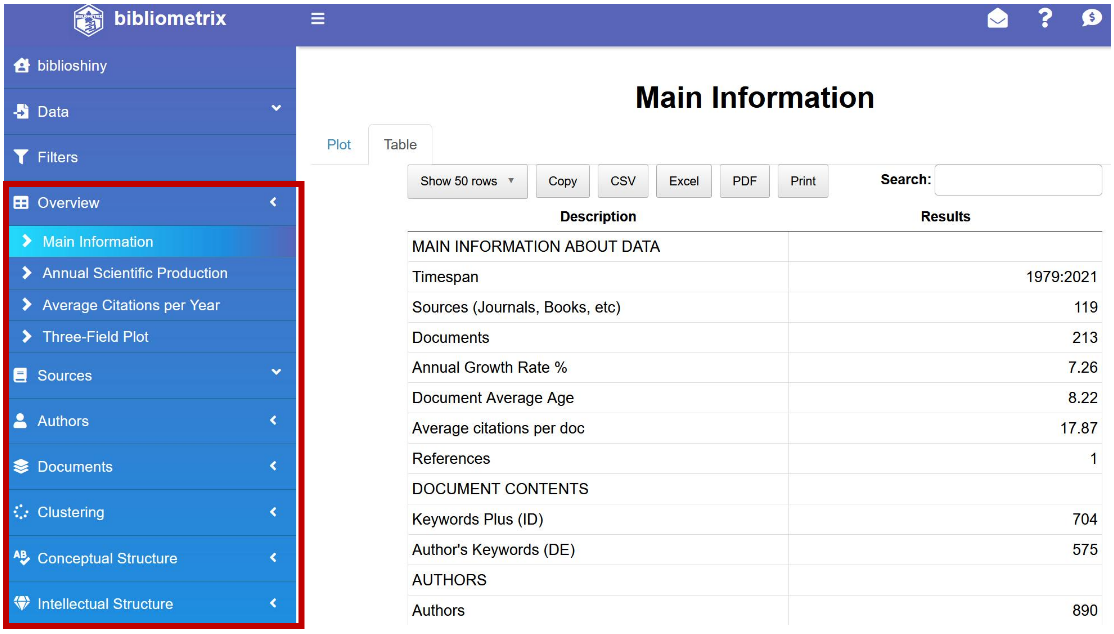

Fig. 3 Key messages from related publications

Step 5: The drop-down menu bar of each theme contains detailed information. The two images below show the drop-down options for the " Authors " and " Documents " menu bars respectively. All images and tables can be downloaded directly from this

page.

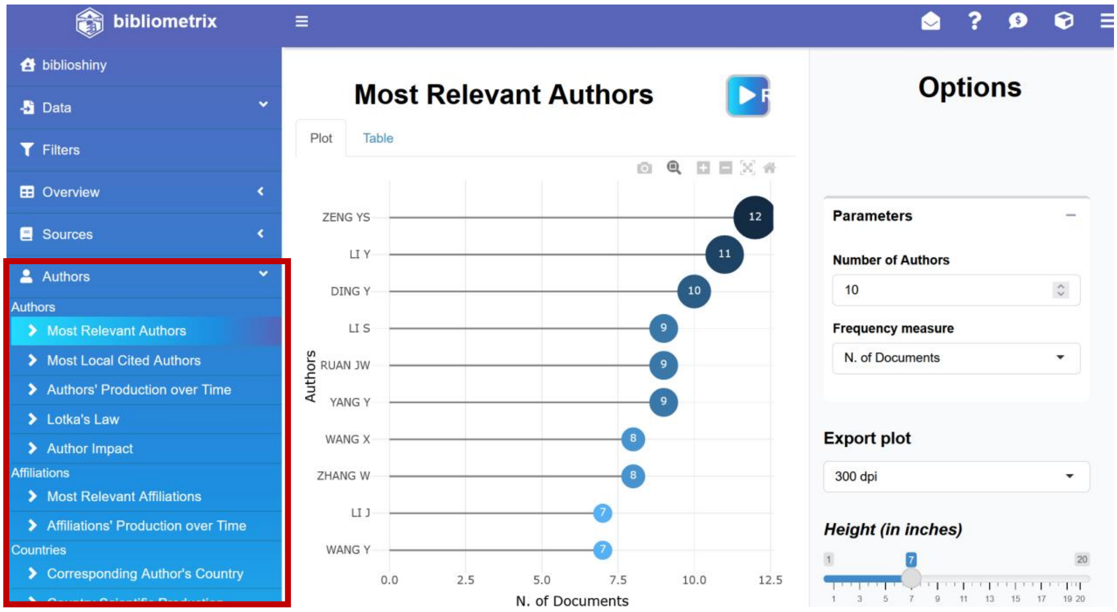

Fig. 4 Information about the "Author" menu bar

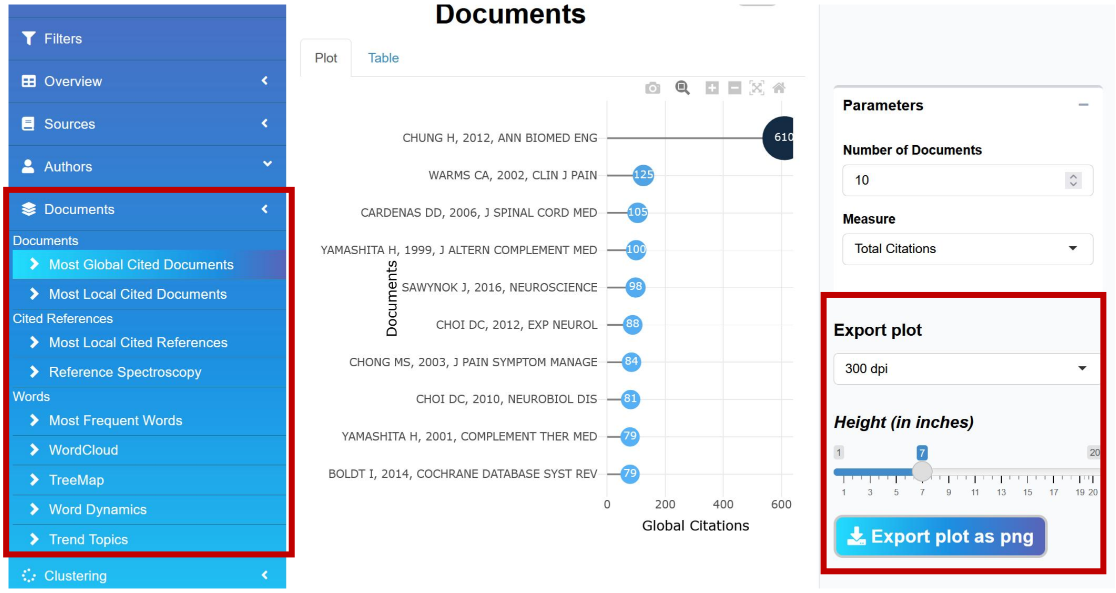

Fig. 5 Information about the "Documents" menu bar

Step 6: Perform quantitative analysis on the exported publication data.
